# A Multifunctional Characterization Test Bench for Shape Memory Alloy Micro-Wires—Design, Implementation and Validation

**DOI:** 10.3390/ma16134820

**Published:** 2023-07-04

**Authors:** Dominik Scholtes, Marvin Schmidt, Philipp Linnebach, Stefan Seelecke, Paul Motzki

**Affiliations:** 1 Intelligent Materials Systems Lab, Center for Mechatronics and Automation Technology, ZeMA gGmbH, 66121 Saarbrücken, Germany; stefan.seelecke@imsl.uni-saarland.de (S.S.); paul.motzki@imsl.uni-saarland.de (P.M.); 2 Intelligent Materials Systems Lab, Department of Systems Engineering, Department of Materials Science and Engineering, Saarland University, 66123 Saarbrücken, Germany

**Keywords:** shape memory alloy, nickel–titanium, NiTi, Nitinol, material characterization, actuator, superelastic, tensile test, high temperature, hardware-in-the-loop

## Abstract

Most relevant to predicting the behavior of shape-memory-alloy (SMA)-based actuator-sensor applications activated by Joule heating are the electro-mechanical characteristics of the material under consideration. For a comprehensive characterization, a single setup that is able to provide all relevant data and information is desirable. This work covers the design, implementation and validation of such a high-end test bench for the holistic characterization of SMA micro-wires. In addition, the setup provides the possibility of application simulation experiments. Key elements of the design are the clamping mechanism guided on air bearings, a linear direct drive, a high-resolution load cell, a high-precision constant current source and a stress-controlled in-line wire sample installation. All measurements take place inside an isolated, temperature-controlled chamber. With the presented setup, the electro-mechanical and thermal characteristics of SMA wire samples with diameters from 20 µm to 100 µm can be determined. Via hardware-in-the-loop (HiL) implementation, the outputs with different biasing mechanisms and additional end-stops can be simulated even at high ambient temperatures. The generated results facilitate the prediction of the exact characteristics of SMA-driven actuator-sensor systems in a variety of applications and lead to a better general understanding of the alloy’s properties. All functionalities and features of the setup are presented by discussing the results of exemplary experiments.

## 1. Introduction

Shape memory alloys (SMA) show, on the one hand, a great potential to reduce the weight, size and energy consumption of existing drive systems. On the other hand, they enable the integration of actuators in new areas, where spatial conditions typically prevent active elements from being implemented.

From an engineering perspective, SMA wires can be seen as electrical actuators in wire shape, and from a material science view, their thermo-mechanical behavior is of foremost interest, which is the reason for which much research concerning the thermo-mechanical behavior of SMA materials is being conducted. However, for the design of new SMA-driven systems, measurement data based on electrically heated wires and the characteristic of their electrical resistance are of high relevance. Based on this, the temperature dependency and, especially, methods to influence the transition temperatures of the material are of concern to investigate the limitations of SMA-driven actuators.

The fundamentals of thermal SMAs have been discussed extensively in research articles over the last decades [1,2,3,4,5]. Hence, in this work, the following paragraph gives only a short summary on the topic.

SMAs have the highest known energy density as an actuator and combine this with resistance-based self-sensing capabilities [6,7,8]. They are often used in the form of wires and are commercially available in the form of binary nickel–titanium (NiTi). This is also called Nitinol. Nitinol was first investigated by researchers of the U.S. Naval Ordinance Laboratory in 1963 [9]. Depending on the alloy composition, the material shows varying thermo-mechanical behavior. Ni-rich NiTi is superelastic and can be stretched up to 10% at room temperature without permanent damage [10]. The Ti-rich variant, on the other hand, undergoes a (quasi-)plastic deformation if stretched at room temperature. When it is then heated to the so-called transformation temperature, it transforms back to its original geometry. This behavior is called shape memory effect, and strains of 5% or more can be fully recovered [3]. Both effects are based on a reversible rearrangement of the materials’ crystal lattice structure, in which a phase transformation from martensite to austenite takes place. The composition of the lattice structure depends on the temperature and the material stress, which is why we talk about the thermo-mechanical behavior of NiTi [11]. To use SMAs as actuators, a Ti-rich NiTi wire is typically heated either by electrical power via Joule heating or passively by a high-temperature fluid in contact with the alloy [12,13]. Because of their high energy density, SMA wires are especially suitable for small and lightweight actuator systems, such as valves, small-size gripping systems and optical image stabilization [14,15,16]. Other fields of research include continuum robots for catheters and endoscopes as well as bionic applications [17,18,19,20]. In all these applications, the self-sensing feature of SMA wires is put to use, and externally positioned sensors are dispensable. The sensing is based on a change of the electrical resistance that can be observed when the SMA undergoes the austenite–martensite transformation. The resistance depends on the contemporary crystal structure, the SMA’s length and cross-sectional area, as well as its temperature [12,21].

SMA actuators in the shape of thin wires have many benefits and are therefore the focus of this work, just as they are also the focus of many commercially available products and recent research and developments [14,22,23,24,25]. Due to their unique form factor, they create much freedom for design, and their surface-to-cross-sectional area ratio enables faster cooling compared to other forms. The implementation of these wires and electrical contacting are already well-understood, and they can be bundled to create muscle-like strands that exhibit high forces with unchanged dynamics [15,26,27]. As most technical applications have high requirements on the dynamics of the system, this work concentrates on wires with diameters of 100 µm and below, which are also referred to as microwires. These microwires have been shown to realize switching cycle times of 1 Hz to 35 Hz with natural convective cooling in air [2,28].

Dynamic mechanical analyzers (DMA) are used for the thermo-mechanical characterization of SMA wires, but they lack the electrical components necessary to characterize Joule-heated actuator wires as well as the required repeatable installation process [29,30]. To achieve significant and repeatable measurement data with which the behavior of Joule-heated SMA wires can be systematically investigated, a test bench is required that is designed to conduct a variety of experiments on a single sample. The goal of this work is the development and implementation of a fully adjustable setup for actuator simulations even at high ambient temperatures, on which tensile tests and SMA wire training procedures can also be performed. Fundamental measurements form the basis of SMA wire characterization; these enable the systematic comparison of different wire samples concerning their alloy composition and heat treatment, among other things. This enables the selection of the best specimen for a certain application and helps to increase the overall understanding of SMA wire behavior. The data are also used to parametrize and validate numerical SMA models and simulations, as was undertaken by Mandolino et al. with measurement data acquired on the setup described in this work [31]. For the design of sophisticated SMA-based actuator systems with uncommon load scenarios (e.g., high ambient temperatures, high material stress), it is important to investigate the SMA’s characteristics under the exact same conditions as they are in the application. This ensures that the system works exactly as intended.

The remainder of this paper is structured as follows. Section 2 covers the design and features of the test rig as well as the control and measurement system. In Section 3, all functions of the setup are validated and discussed on the basis of exemplary experimental results, and Section 4 finishes with a conclusion and an outlook for future publications.

## 2. Mechanical Design of the Test Bench, Data Acquisition and Measurement Setup

In this section, the mechanical design of the characterization setup is described in detail. All parts and functions are discussed, including the measurement setup and data acquisition. The goal is to create a test bench on which a multitude of experiments can be conducted on a single test specimen and on a single platform without manual manipulation of the SMA wire. Significant results as well as the ability to change the specimen in an easy way and to reinstall it with the highest repeatability are mandatory.

**Figure 1:**
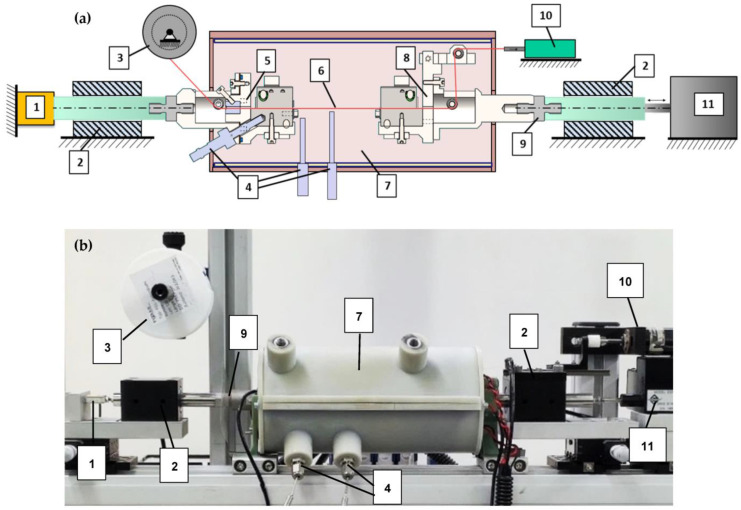
(**a**) Schematic design of the experimental setup. (**b**) Picture of the fully implemented test rig with closed heating chamber including the following components: 1: load cell; 2: air bearing; 3: SMA wire reel; 4: PT100 sensors; 5: fixed wire clamp (F); 6: SMA wire; 7: heating chamber; 8: moving wire clamp (M); 9: insulation adapter; 10: preload drive (P); 11: linear direct drive.

The setup is developed for wires with a diameter of 20 to 100 µm and a length of about 100 mm. Due to the delicacy of SMA microwires, a multitude of measurements have to be taken into account to achieve repeatable and significant experimental results. A schematic of the setup design and a photograph of the implemented test rig are presented in Figure 1.

**Figure 2:**
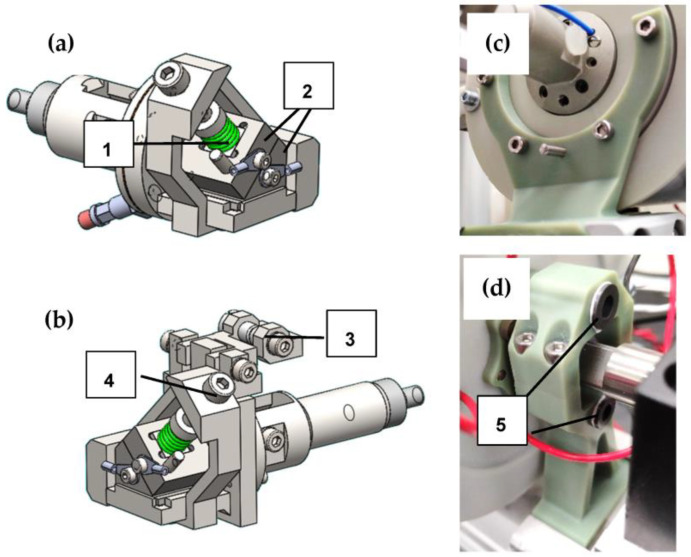
On (**a**,**b**), the CAD designs of both wire clamps are displayed. Image (**c**) shows the fixed bearing of the isolating chamber, and (**d**) illustrates the floating bearing, which allows for a thermal expansion. The following parts are displayed: 1: spring to load the clamps, 2: clamping jaws, 3: guiding rollers for the SMA wire, 4: bolt to manually tighten clamps, 5: linear guides for thermal expansion of isolating chamber.

**Figure 3:**
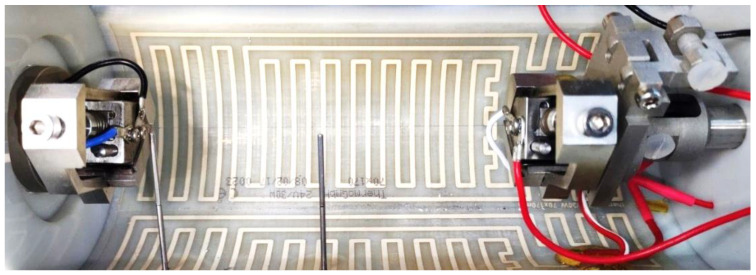
Picture of the inside of the chamber with heating foil, both wire clamps with a SMA wire sample installed, electrical wiring and PT100 sensor tips.

From a mechanical point of view, the key element of the whole setup is the clamping mechanism to fix the SMA wire specimen. The CAD assembly of the clamps can be seen in Figure 2a,b. These clamps fix the SMA wire mechanically, and all electrical supply and measurement connections are attached, as can be observed in the picture of the implemented clamps in Figure 2 and Figure 3. The clamps are an assembly of milled parts made from stainless steel. Two offset angled surfaces create a V-shape that defines the position of the SMA wire exactly in the center of the clamps and in line with the whole setup. The wire is clamped on the inner surface of the V-shape over a length of 10 mm. The clamps are tightened manually by spring-loaded hexagonal bolts (parts 1 and 4 in Figure 2). The spring is designed such that the required clamping force of the SMA wire is reached when it is fully compressed. Thus, the clamping force is repeatable, and the wire is held in place safely. Furthermore, the clamps feature guiding rollers and small holes to guide the SMA wire in and out of the isolating chamber they are placed in. They are electrically isolated from the rest of the test rig by PTFE adapters (Figure 1, part 9) in order to enable the electrical heating of the SMA wire without any short circuits.

Each clamp is mounted to an air bearing shaft, as shown in Figure 1 (2), with the goal of reducing friction losses as much as possible. This is crucial for meaningful micro-wire force measurements, due to the low absolute force level (0.1 N at 200 MPa for a 25 µm diameter wire). The bearing is also necessary to keep the lateral forces caused by the weight of the clamps from affecting the force measurement and the linear drive.

The fixed wire clamp (F) is connected via the air bearing to a “Futek LSB200 2 lbs” load cell with a measuring range of approx. +/−10 N. Knowing the SMA wire diameter, the material stress can be calculated with the force measurement data. The moving wire clamp (M) is attached to an “Aerotech ANT-25LA” linear direct drive. Its range of travel is 50 mm, and it is equipped with a sub-micrometer-resolution position encoder that is used as feedback to obtain the wire strain. Additionally, these two components set the base for a closed-loop force and position control, called hardware-in-the-loop (HiL). To ensure a consistent specimen length of 100 mm for each experiment, the distance between the clamps is precisely set using a gauge, and the linear drive is “homed” and in fixed-position mode. To install the SMA wire in a repeatable way, an additional stepper motor (Figure 1, part 10) is installed to pull the wire to a defined pre-stress before it is fixated with the clamps. This process will be discussed in detail in Section 3.

Microwires need to be shielded from irregular air flow and variable convection. Slight air streaming in a room, which occurs, for example, due to the air condition, influences the wire temperature and leads to irregular measurement results. For this reason, the developed setup features a cylindrical chamber with a diameter of 90 mm and a length of 200 mm, in which the wire sample as well as the clamps to fix it are placed. The upper half of the chamber (part 7 in Figure 1b) can be removed to access the wire and the clamps. Inside, a heating foil is attached to the chamber’s walls, which is depicted in Figure 3. In combination with PT100 temperature sensors, the temperature within can be precisely controlled and monitored to set points of up to 100 °C. To account for the expansion of the material when the chamber is heated, the mounting points are located close to the axial center of the cylinders faces, including a floating bearing on one end. With these measures, which are depicted in Figure 2c,d, blockage of the moving clamps due to thermal expansion of the chamber is prevented, and an annular gap for the clamps of less than 0.5 mm is set.

To achieve the highest possible repeatability and least handling effort, the wire is taken directly from the reel on which it is delivered by the supplier. It is guided into the chamber through the open fixed wire clamp (F), onto the featured guiding rollers, and then it exits on the opposite side, where it can be mounted to the pre-loading system (P) (Figure 1, part 10). This procedure is only necessary for the initial setup. After that, a fresh sample is installed by pulling a new part of the wire from the spool to the experimental chamber. This kind of controlled installation process has the benefit that no manual handling or cutting of the SMA wire is necessary to install a new specimen after the first setup.

**Figure 4:**
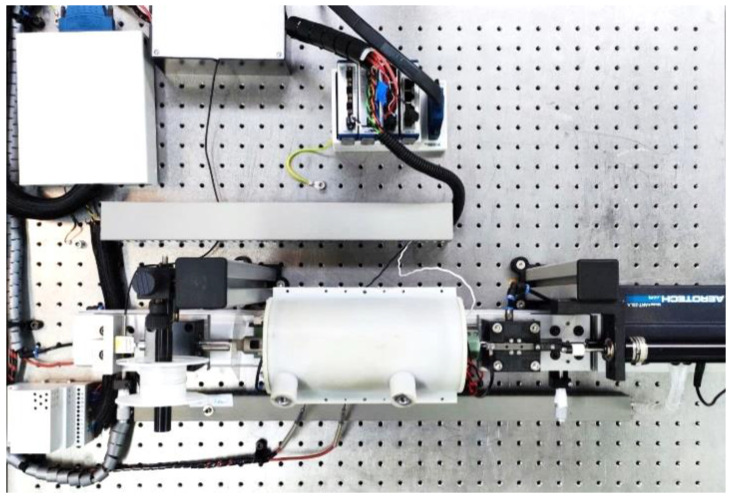
Top-view picture of the whole setup installed on an air damped table, including measurement equipment.

The whole setup is mounted on a “ThorLabs” rail system (Figure 4) combined with milled custom adapters and positioning stages for a precise alignment of the setup. The outer measurements of the mechanical setup are 800 mm × 350 mm × 200 mm (l × h × w). The setup is installed on an optical table with air damping to keep shocks and vibrations in the environment of the setup from interfering with the highly sensitive force measurement.

A “National Instruments NI PXI7852R” FPGA-based system and “LabVIEW” programming environment on a PC serve for the control unit as well as data acquisition. The system features several high-resolution analog and digital in- and outputs. The measured values are as follows: position of the moving wire clamp, force of the SMA wire, voltage and current of the wire, temperature inside the chamber. With these values, we can retrace the stress, strain, electrical resistance and electrical power of the SMA wire specimen. The ambient temperature in the heated chamber is measured with two PT100 sensors. They are placed in proximity to the wire: one is close to the fixed clamp, and the other is in the center of the chamber, as can be seen in Figure 3. Another temperature sensor can be added to measure either the temperature inside the fixed clamp or the ambient temperature of the room in which the setup is placed.

A temperature measurement of the wire specimen itself is omitted. A reliable and meaningful measurement can only be performed with a contactless measurement method, for example, an infrared camera. A sensor in physical contact with the wire works as a heat sink, which corrupts the result and does not acquire the temperature distribution over the wire length. Due to the necessary calibration, emission factors, curved wire surface, the small diameter of the SMA and the fact that the sample is enclosed in the chamber, an infrared measurement also proves to be challenging.

Feedback control loops are implemented on the FPGA real-time system for the temperature in the chamber, the electrical power to heat the SMA wire, as well as the force and position of the linear direct drive. With the latter, so-called HiL tests can be performed, which enable one to simulate a spring or constant load as a biasing element of the actuator wire. Position control modes enable the addition of arbitrary end-stops to the load scenarios.

Apart from heating the ambient air in the chamber, the wire is heated via Joule heating. Therefore, a custom current source with 24 V supply voltage is designed. An output current of 0–250 mA is set and controlled by a 0–10 V input signal from an NI module. It features three separate 0–10 V output channels with a different measurement range to measure the supplied current. For high electrical powers, in austenitic tensile tests, for example, the 0–250 mA channel is used. For acquiring precise resistance values close to zero power in the martensitic state, for ambient heating and when cooling down in HiL tests, the channels 0–25 mA and 0–6.25 mA are used for a better resolution. The current source is validated in preliminary work, and an absolute measurement error of 10 µA is achieved. This kind of precision is necessary to receive significant resistance values.

All input parameters for the experiment are set on a user interface on the PC, where the experiments are started, and the measurement data are depicted. When an experiment is finished, the data are saved for post processing, plotting and analysis with “MATLAB”.

## 3. Result and Discussion of Validation Experiments and Functionalities

In this section, the functionalities and features of the test rig are proposed and validated with exemplary results on a variety of NiTi wire specimens. The full range of possible experiments and sample diameters is introduced by means of various wire manufacturers. The authors do not intend to compare the different manufacturers and companies to each other, which is why each experiment is performed with a number of varied parameters to place the focus on the capabilities of the test setup. The goal here is to present the options that are provided by the results generated with the help of the test rig, to evaluate and extract various parameters of interest and application relevant data.

**Figure 5:**
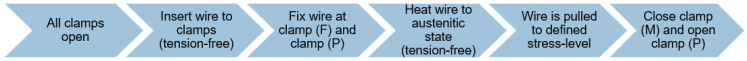
Flowchart of the installation procedure of a SMA wire specimen in the test rig.

Before each experiment, the test specimen needs to be installed in a precise and repeatable manner. As described in Section 1, the length of a SMA actuator is defined by its current temperature and stress as well as the stress-temperature history. In this article, we define the austenitic length of the SMA wire at 10 MPa stress as “zero length” L_0_. The required heating power can easily be acquired in preliminary experiments, by testing at which power level the specimen does not contract any further. The benefit of setting this length as zero is that a SMA wire cannot become any shorter than when it is 100% in the austenite phase, which makes it absolutely repeatable. The stress is chosen, so that the wire is straight without slack or bending, and the correct length is set. For a repeatable and defined length and crystal lattice composition of each SMA wire specimen to be tested, the installation procedure, presented in a flowchart in Figure 5, is performed.

After all clamps are opened, the specimen is mounted to the fixed wire clamp (F) and the preloading drive (P) outside the chamber (Figure 1). The moving wire clamp (M) stays open, and the wire is only guided through it. The wire is pulled by the preloading drive, while it is electrically heated above A_f_ temperature, until the defined stress of 10 MPa is reached. Then, the bolt of clamp (M) is tightened to fix the specimen, and the installation process is finished. The heating current is switched off, and clamp (P) is opened. After this process, the SMA wire sample is known to have a L_0_ of 100 mm, and it is ready for the following experiments.

Each experiment is started by conducting a so-called “reset test”, with the goal of having the same repeatable initial condition for every test and reducing the influence of the order in which different experiments are conducted on a specimen. To reset a wire sample to these initial conditions, it is heated to austenite and cooled back down to martensite under stress-free conditions.

In general, two different sorts of experiments can be conducted on the test rig: tensile tests, in which the wire is cycled mechanically, and actuator tests, in which the wire is cycled thermally via Joule heating. Both variants can be repeated any number of times, which is used for training experiments. SMA samples for tensile tests are heated with constant electrical power, constant current or via the ambient air temperature. The parameters that are variable are the maximum strain, the strain rate and the level of heating power/current/temperature. The actuator tests are conducted with the help of HiL. Biasing elements such as springs and masses can be simulated with the help of a closed-loop force and position control of the linear direct drive. The heating is achieved via triangular current signals, as a constant power supply is not common in actuator applications due to the complexity. Parameters that can be varied are the biasing force and spring stiffness, the activation signal duration, the maximum heating current and the ambient temperature. To limit the travel, as it is also often done in applications, arbitrary end-stops can be set.

The following resulting data are extracted from all experiments: time in s, force in N, position (of the linear drive) in mm, voltage over the SMA in V, current in A, ambient temperature in °C. The diameter of each wire sample is measured in a cold state with a micrometer gauge to calculate the material stress. The change in the wire’s cross-sectional area under strain or contraction is not considered. 

In the following subsections, only stress–strain diagrams are presented instead of force elongations graphs, to have a common and comparable basis for varying diameters or wire lengths. Force and elongations can easily be calculated, as the necessary parameters are listed for each experiment. Electrical resistance and power are calculated from the voltage and current measured during the experiments.

### 3.1. Validation of Installation Process

**Figure 6:**
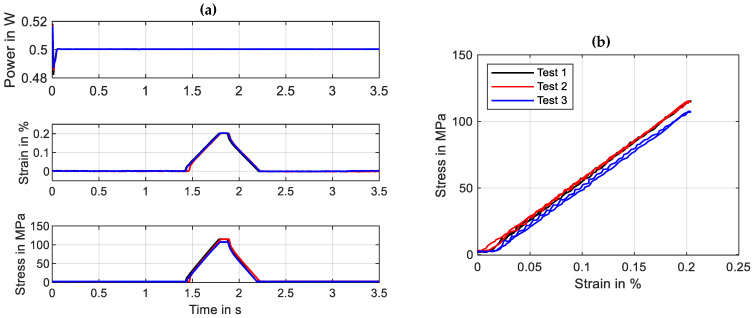
Results of the validation experiments for the wire installation process. Depicted are input and output parameters over time in (**a**) and stress over strain in (**b**). Test parameters: tensile test, max. strain 0.2%, strain rate 0.005 s^−1^, wire specimen “SAES Getters Smartflex” 100 µm, electrical heating power 0.5 W, 3 iterations.

To validate the previously described installation procedure of wire samples, a special test series is conducted. The goal is to check the repeatability of the procedure, so that wire samples can be changed, and the next experiment will have the same initial conditions as the one before. For the checkup, a “SAES Getters Smartflex 100” NiTi wire specimen with a measured diameter of 100 µm is used [32]. A specimen is installed in the defined manner, and a tensile test with 0.2% maximum strain and a constant power of 0.5 W is performed. The installation procedure and the following tensile test is repeated three times, of which the results are presented in Figure 6.

The results of the validation experiment show that the L_0′_ has a maximum variance of 0.015% strain, resulting in 7 MPa difference in stress at 0.2% strain. These values prove that a good repeatability of the installation process is provided.

### 3.2. Tensile Tests under Constant Electrical Power Heating

**Figure 7:**
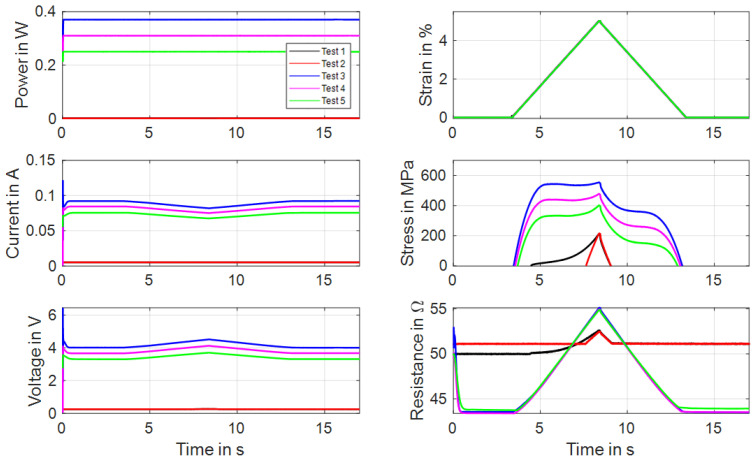
Results of tensile tests with wire specimen “Dynalloy Flexinol LT (70 °C)” of 49 µm diameter under varied constant power. All results are plotted over time. Test parameters: max. strain 5%, strain rate 0.01 s^−1^, ambient temperature 22 °C.

The first function of the test rig to be introduced are tensile tests in which the maximum strain, strain rate and heating method are variable parameters. The results are used for a basic characterization of actuator wires and allow for evaluation and comparison, for example, of different alloy compositions and transformation temperatures. For this exemplary experiment, the wire is heated with a constant electrical power and stretched to 5% strain with a strain rate of 0.01 s^−1^. A “Dynalloy Flexinol LT (70 °C)” NiTi wire with a measured diameter of 49 µm is used [33]. To examine the tensile characteristics under different conditions, five tensile tests are performed in a certain order. Before the experiment starts, the sample undergoes the previously introduced reset procedure, in which it is heated to full austenite and cooled down without tensile stress. The first tensile test is conducted with no power applied, but 5 mA of measuring current is used to calculate the resistance. This test is repeated once. Then, three more tensile tests are performed with high power (0.37 W), medium power (0.31 W) and low power (0.25 W). For evaluation, the acquired data are plotted over time and can be seen in Figure 7. In between each of these iterations, the reset procedure is omitted.

**Figure 8:**
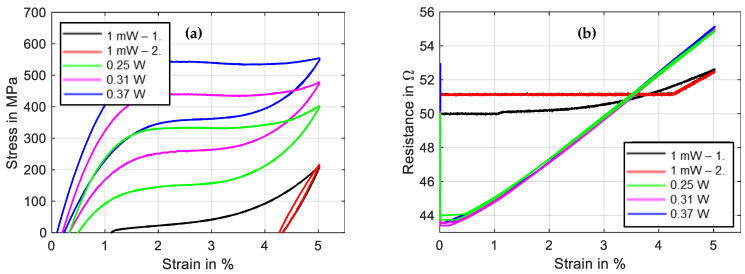
Results of tensile tests with wire specimen “Dynalloy Flexinol LT (70 °C)” of 49 µm diameter under varied constant power: (**a**) shows stress vs. strain, and (**b**) shows resistance vs. strain.

The first two tests, in black and red color, show the martensitic material characteristics. In the first experiment, a detwinning of the martensite can be observed that builds under stress-free cooling from austenite to martensite. After the material is detwinned, the second tensile test shows the purely elastic behavior of martensite. In Figure 8, the results of stress and resistance are plotted over strain. Here, the martensitic detwinning and the elastic branch can be observed as well. In Figure 8a, the typical stress–strain hysteresis with varying transformation plateaus depending on the heating power is recognizable, and in Figure 8b, a linear change in resistance under a tensile load with constant heating power is evident.

### 3.3. Tensile Tests under Constant Electrical Current Heating

**Figure 9:**
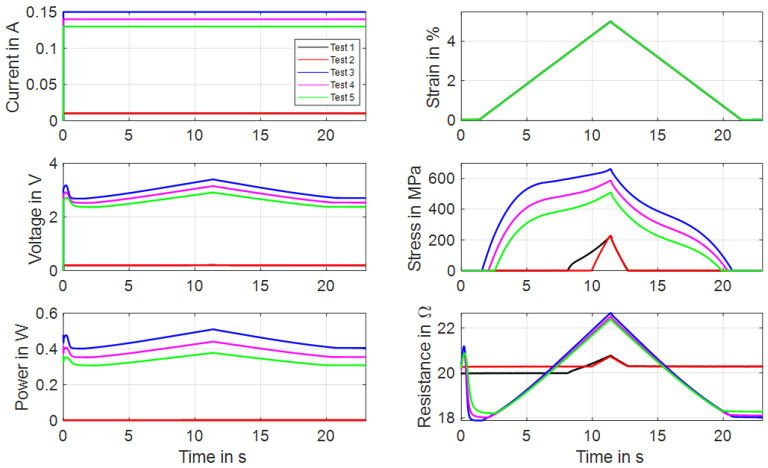
Results of tensile tests with wire specimen “Fort Wayne Metals NiTi #5” of 73 µm diameter under varied constant current heating. All results are plotted over time. Test parameters: max. strain 5%, strain rate 0.005 s^−1^, ambient temperature 22 °C.

**Figure 10:**
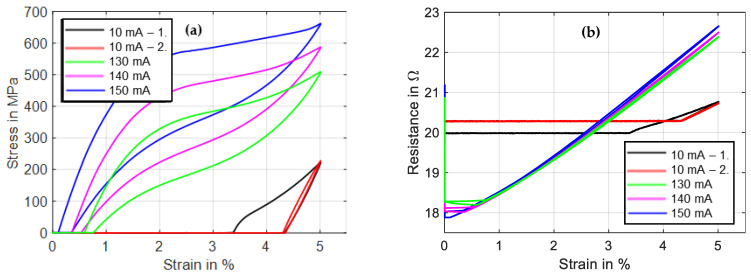
Results of tensile tests with wire specimen “Fort Wayne Metals NiTi #5” of 73 µm diameter under varied constant current heating: (**a**) shows stress vs. strain, and (**b**) shows resistance vs. strain. Test parameters: max. strain 5%, strain rate 0.005 s^−1^.

A power-controlled electrical supply is uncommon in SMA applications due to the complexity and lack of benefits. For scientific tests, on the other hand, this is of interest, because a constant heating power equals a constant temperature if the strain rate and the rate of the change in power are sufficiently small [34]. As the main target of the setup is to provide measurement data that are of relevance for applications, experiments with constant current to heat the wire are also part of the test portfolio and are presented in the following. In the example depicted in Figure 9 and Figure 10, a strain rate of 0.005 s^−1^ at a maximum strain of 5% is chosen for a “Fort Wayne Metals NiTi #5” wire with a measured diameter of 73 µm [35].

The method of the experiments is the same as mentioned in Section 3.2, and the heating is controlled with a constant current instead of constant power. The first tensile test is conducted with 5 mA of measuring current to calculate the resistance. This test is repeated once. Then, three more tensile tests are performed with high current (150 mA), medium current (140 mA) and low current (130 mA). As can be observed in Figure 10a, the main difference from constant power heating is that the slopes of the transformation plateaus are steeper and less distinct, due to the increase in electrical power with increasing wire resistance. A detwinning of the martensite is also evident in this sample for the first tensile test. The amount of twinned martensite and, with it, the intrinsic two-way-effect of a SMA wire are varied in between samples. This depends on individual load history as well as the training and the alloy composition [36]. With the help of the austenitic and martensitic branch of the stress–strain curves in Figure 10a, as well as the resistance–strain graph in Figure 10b, the first important tool for the design of a SMA actuator is available. 

With these results, the maximum stroke and the force output of an actuator with a certain bias element can be estimated by a graphical/geometrical comparison of the austenite and martensite branches. The necessary electrical specifications can be estimated with the maximum and minimum values of austenitic and martensitic resistance values and the equivalent heating currents.

### 3.4. Tensile Tests under Ambient Temperature Heating

**Figure 11:**
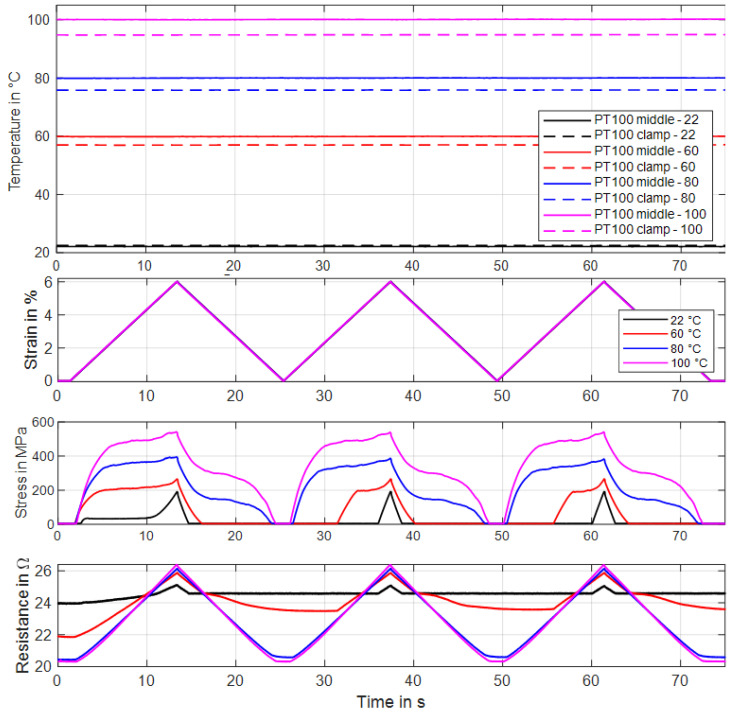
Results of tensile tests with a wire specimen by “Ingpuls GmbH” of 73 µm diameter with varied ambient temperature. Three iterations are performed for each temperature. All results are plotted over time. Test parameters: max. strain 6%, strain rate 0.005 s^−1^, measurement current 10 mA.

**Figure 12:**
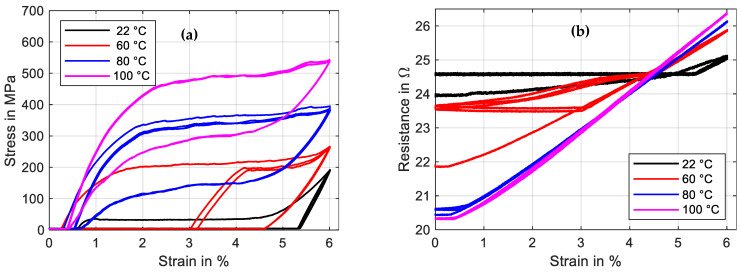
Results of tensile tests with a wire specimen by “Ingpuls GmbH” of 73 µm diameter with varied ambient temperature. Three iterations are performed for each temperature. (**a**) shows stress vs. strain, and (**b**) shows resistance vs. strain. Max. strain 6%, strain rate 0.005 s^−1^.

The chamber that isolates the specimen from the surrounding air can be used to passively heat the SMA wire, as described in Section 2. Therefore, a certain temperature setpoint is chosen, and the inside of the chamber is heated up to that level. In this subsection tensile tests with a passively heated wire are proposed. The outputs are used to check at which temperature a sample starts to build up austenite phase fractions and to compare electrically heated experiments with temperature-controlled tests for evaluating transition temperatures. For these tests, a NiTi wire sample provided by “Ingpuls GmbH” with a diameter of 73 µm is used [37]. For each temperature setpoint (22 °C, 60 °C, 80 °C, 100 °C), a tensile test with three iterations is performed. Before every set of tensile tests, the reset procedure is conducted. Thus, the first iteration of one set deviates from the second and the third, as can be observed in Figure 11 and Figure 12. For the tensile tests of this subsection, the maximum strain is set to 6% with a strain rate of 0.005 s^−1^. All tests are conducted with the same sample in order of rising temperature. The temperature control is based on the PT100 sensor placed in the middle of the chamber, and the measurement in proximity to the clamp provides information about the uniformity of temperature distribution inside the chamber. The temperature-over-time graph in Figure 11 makes this distribution visible. Meanwhile, the air in the middle of the chamber is steady at the chosen setpoint. The temperature at the edge is slightly lower due to small air gaps in the surrounding and the clamp working as a heat sink. 

The shape of the stress–strain hysteresis in Figure 12a, for temperatures of 80 °C and higher, shows the typical austenitic characteristics, as can be expected for an actuator wire. For the experiment at room temperature, the same detwinning effect in the first cycle as in the previous subsections can be observed. The set of tensile tests at 60° C ambient temperature shows that the material is already partially transformed to austenite. To achieve the resistance values, a measurement current of 10 mA is applied during all experiments. The results of the resistance measurements are plotted over strain in Figure 12b. The typical linear behavior is observed at 22 °C, 80 °C and 100 °C. The 60 °C experiment shows a hysteretic characteristic, as the sample seems to partially transform back to austenite when it is fully relaxed.

### 3.5. Actuator Tests with Constant Load Bias

**Figure 13:**
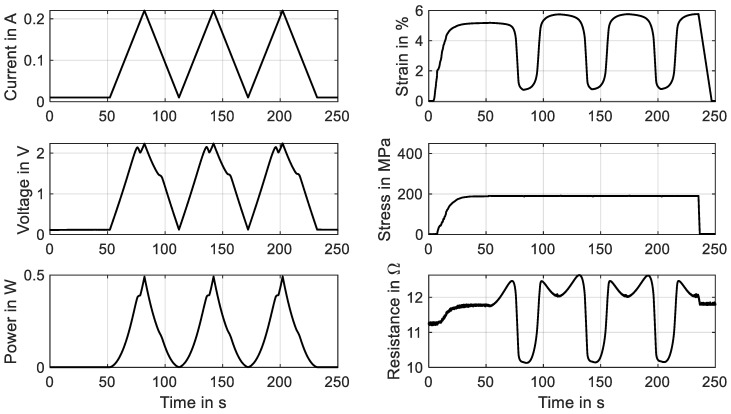
Results of actuator tests with wire specimen “SAES Getters Smartflex” of 100 µm diameter under constant load biasing of 188 MPa. A triangular current signal with an amplitude of 220 mA and a signal duration of 60 s are applied. Three iterations are performed for the experiment, of which all results are plotted over time.

The so-called HiL function of the test rig enables the performance of actuator tests with the simulation of various biasing mechanisms. The linear direct drive is used in a force-control mode with a closed loop feedback of the force measurement provided by the loadcell. Constant load biasing with arbitrary levels, a variable linear bias spring with an adjustable force offset and freely selectable end stops are implemented. In this subsection, the constant load biasing function of the test rig is proposed with the example of a 100 µm “SAES Getters Smartflex” NiTi wire. The time sequence of the output data is depicted in Figure 13.

**Figure 14:**
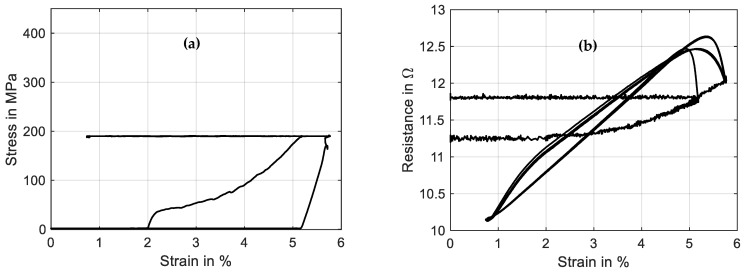
Results of actuator tests with wire specimen “SAES Getters Smartflex” of 100 µm diameter under constant load biasing of 188 MPa. A triangular current signal with an amplitude of 220 mA and a signal duration of 60 s is applied. Three iterations are performed for the experiment. (**a**) shows the stress vs. strain behavior, and (**b**) shows resistance vs. strain.

The test sequence is started with only the measurement current of 10 mA applied to achieve continuous resistance measurement results from the experiment’s start. The linear drive is moved to pull the wire to the preset force value of 1.48 N, which results in a material stress of 188 MPa. When the setpoint is reached, a triangular current signal with a maximum value of 220 mA and a signal duration of 60 s, as displayed in Figure 13, is run three times. This results in the wire contracting to a strain of 0.7% and releasing back to 5.7% of absolute strain. A difference between the initial cycle and the next cycles can be observed in the strain and stress signal in Figure 13 as well as in Figure 14a. The reason for this behavior lies in the detwinning of martensite in the first cycle. In the reset procedure that is performed before each set of experiments, martensite in a twin structure is generated because of the “load-free” condition in the experiment. In the last two cycles of the experiment, the biasing stress only allows for the formation of detwinned martensite.

This difference due to the initial lattice structure of the NiTi is also observed in the resistance-over-strain graph in Figure 14b. The linear correlation between resistance and strain in tensile tests gives way to a hysteretic behavior. Taking into account the start of the second signal, when the behavior is stabilized, the resistance rises from 12 Ω to 12.7 Ω as the wire temperature rises, and only a minor change in geometry and the crystal lattice appears. In the phase transformation to austenite, accompanied by a contraction of about 4.8%, the resistance drops to 10.2 Ω. As the heating current is reduced again, a slight dip in resistance can be observed, which leads to the first loop in the hysteresis. This is due to the temperature outweighing the influence on the resistance. Then, the crystal lattice transforms back to martensite, accompanied by a rise in resistance of up to 12.5 Ω. Another loop is formed in the cooling process, as the slope of the resistance over strain is not monotonous and cuts the heating branch of the resistance–strain curve. Returning to full martensite and room temperature, the starting value of 12 Ω is reached again.

**Figure 15:**
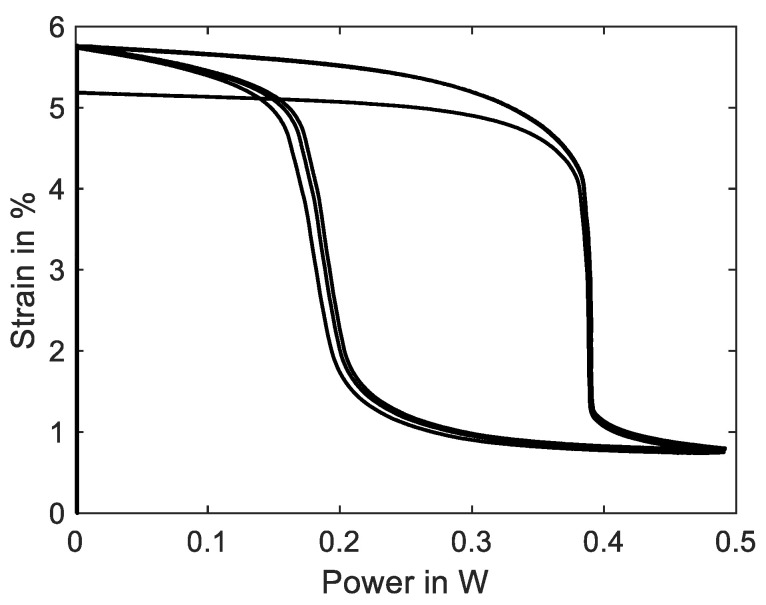
Results of actuator tests with wire specimen “SAES Getters Smartflex” of 100 µm diameter under constant load biasing of 188 MPa. A triangular current signal with an amplitude of 220 mA and a signal duration of 60 s is applied. Three iterations are performed for the experiment. The diagram shows the strain over electrical power behavior.

The SMA wire is heated electrically and quantitative temperature measurements of micro wires cannot be performed in this test setup and neither can the wire temperature be measured in SMA-driven applications. The power–strain diagram is shown in Figure 15 and can be compared to a typical temperature-over-strain diagram. As the experiment is conducted in a quasistatic manner due to the slow heating rate, a correlation between electrical power and temperature is allowed [8]. The typical shape of a SMA temperature–strain hysteresis can be qualitatively observed in the power–strain plot as well. In systematic experiments, these plots can be used to achieve the austenite start and finish power of a wire sample for various loads. With the help of temperature-controlled experiments, a comparison is possible as well.

A soon as the electrical power is increased, the SMA sample starts to contract with a slow rate. After about 1.5% contraction, at 0.39 W, the main portion of the phase transformation takes place, and the wire contracts suddenly about 3%. The last 0.4% strain is reached at 0.5 W. The transformation back to martensite is less sudden, has a flatter slope and occurs mainly at about 0.2 W. From this hysteresis curve, we can extract the information about austenite and martensite start and finish power values and the width of the hysteresis, which helps to compare and evaluate different SMA samples for specific applications.

### 3.6. Actuator Tests with Spring Load Bias

**Figure 16:**
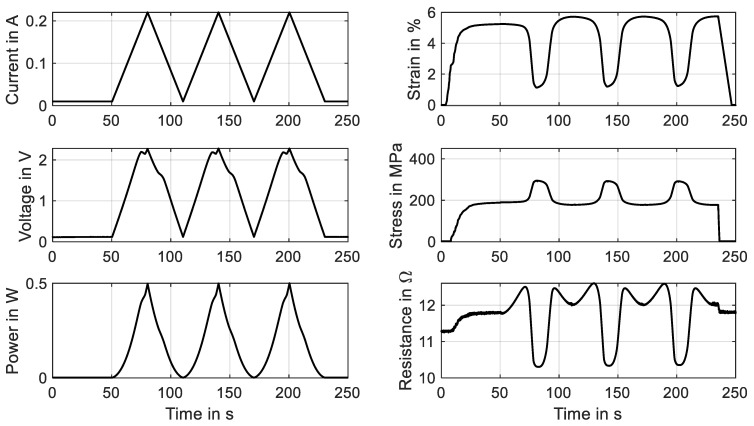
Results of actuator tests with wire specimen “SAES Getters Smartflex” of 100 µm diameter under spring load biasing of 0.2 N/mm and a pretension of 1.48 N. A triangular current signal with an amplitude of 220 mA and a signal duration of 60 s is applied. Three iterations are performed for the experiment, of which all results are plotted over time.

In most cases, the biasing system for an SMA wire in technical applications includes linear springs. The stiffness and pretension of the spring influence the stroke output and resistance of SMA wires. To conduct a systematic study on this influence with variations in the parameters, it is possible to simulate any spring with any pretension with the test setup. Furthermore, we can test and investigate specific actuator configurations in detail before building the actual system. In this example, a “SAES Getters Smartflex” NiTi wire with 100 µm diameter is prestressed to the same level as in Section 3.5, and a spring stiffness of 0.2 N/mm is applied. The wire is heated with the same sequence as before, which is a triangular signal with 60 s duration and an amplitude of 220 mA. The results of three iterations of the experiment are shown in Figure 16.

**Figure 17:**
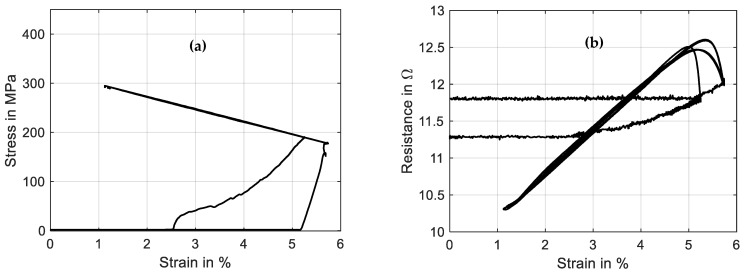
Results of actuator tests with wire specimen “SAES Getters Smartflex” of 100 µm diameter under spring load biasing. A triangular current signal with an amplitude of 220 mA and a signal duration of 60 s is applied. Three iterations are performed for the experiment. (**a**) shows the stress vs. strain behavior, and (**b**) shows resistance vs. strain.

The results of stress and resistance plotted over strain are displayed in Figure 17. The same difference in the initial cycle can be observed as in Section 3.5 due to twinned martensite formed in the reset procedure before the actual experiment. The stress–strain curve follows the spring characteristic and exhibits a stroke of 4.5% in stable conditions after the first activation. The actuator cycles between 170 and 295 MPa. In the strain–time trend of Figure 16, it is observed that the maximum stroke is slightly reduced with ongoing cycling. The SMA behavior is not stable in the high-stress region, and a residual strain builds up.

Comparing the resistance–strain graph in Figure 17b to Figure 14b, the spring-loaded SMA wire shows a less hysteretic behavior but still shows similar crossing loops. This difference can be explained by the stress dependency in the transformation temperatures of the SMA materials. The stress increases with the wire contracting, and at the same time, this increases the transformation temperature. For SMA-driven technical systems, this means that the resistance signal of the self-sensing feature is easier to interpret and correlate with a certain actuator position.

**Figure 18:**
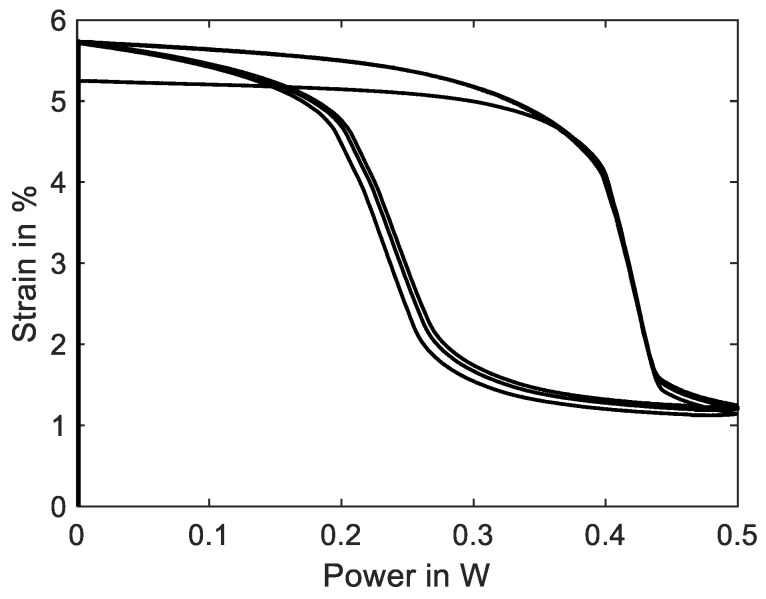
Results of actuator tests with wire specimen “SAES Getters Smartflex” of 100 µm diameter under spring load biasing. A triangular current signal with an amplitude of 220 mA and a signal duration of 60 s is applied. Three iterations are performed for the experiment. The diagram shows the strain vs. electrical power behavior.

The stress dependency of the phase transformation temperature also prostates in the strain–power hysteresis in Figure 18, where the shape of the hysteresis is changed compared to Figure 15. The transformation from austenite to martensite and back is less steep, as phase transformation temperature rises with increasing stress, and vice versa. The higher material stress makes higher heating powers necessary to fully transform the crystal lattice to austenite. By varying spring stiffness and preloading, their influence on heating power and resistance behavior can be studied systematically. The results can help to predict the necessary parameters for high load scenarios of SMA that are rather uncommon and to increase the performance of self-sensing-based control strategies for SMA actuators.

### 3.7. Actuator Test with Spring Load Biasing, End Stops and 60 °C Ambient Temperature

**Figure 19:**
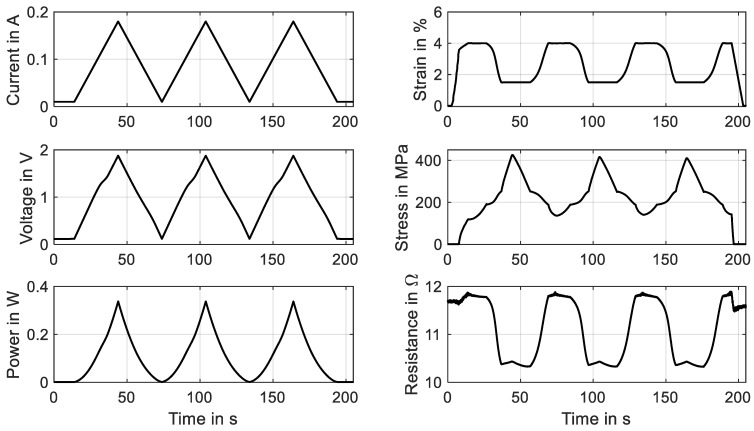
Results of actuator tests with wire specimen “SAES Getters Smartflex” of 100 µm diameter under spring load biasing with end stops and 60 °C ambient temperature. A triangular current signal with an amplitude of 180 mA and a signal duration of 60 s is applied. Three iterations are performed for the experiment, of which all results are plotted over time.

The full potential of the test rig is put on display in this following subsection. For applications in commercial products, the automotive and the industrial environment, the ambient conditions change, and increased temperatures prove to be especially challenging for SMA actuator performance. Additionally, a defined movement of drive units is often supported by end stops. To investigate the capabilities of SMA wire actuators at high temperatures and the influence of the biasing force on the characteristics, several features of the test rig are combined for these experiments. Before the experiment is started, the chamber is heated to a stable temperature of 60 °C. The wire specimen is again a “SAES Getters Smartflex” with 100 µm diameter, which is prestressed to 188 MPa. Due to the increased ambient temperature, the amplitude of the triangular signal of the heating current is reduced to 180 mA to avoid overheating and damaging the sample. To simulate a realistic application, for example, a fluidic valve, two end stops at 1.5% and 4% strain are implemented. The experimental procedure is similar to Section 3.5 and Section 3.6, and the end stops limit the range of travel. The results of all measurement data are presented over time in Figure 19. Temperature is not included, as it is held constant along the experiment.

**Figure 20:**
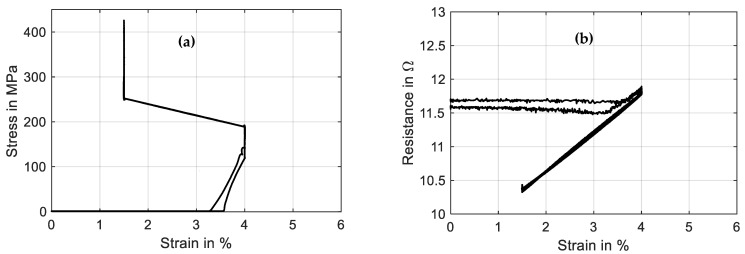
Results of actuator tests with wire specimen “SAES Getters Smartflex” of 100 µm diameter under spring load biasing with end stops and 60 °C ambient temperature. A triangular current signal with an amplitude of 180 mA and a signal duration of 60 s is applied. Three iterations are performed for the experiment. (**a**) shows the stress vs. strain behavior, and (**b**) shows resistance vs. strain.

Due to the end stop at 4% strain, the movement of the SMA sample starts after the 188 MPa spring preload is overcome. This can be observed in a good manner in Figure 20a. As the second end stop is reached at 1.5% strain, the stress rises up to 420 MPa. This SMA sample is not trained for stresses higher than 200 MPa, and therefore, the maximum stress drops with each cycle, as can be observed in Figure 19. The strain output is not affected, as the end stops limit the travel beforehand.

The resistance (Figure 20b) is even less hysteretic than in the previous subsection, because of the ambient temperature being close to the austenite start temperature and the limited travel. Thus, loops at both ends are cut off as well. The bends in the resistance signal in Figure 19 when the end stops are reached promise a good detectability with the help of resistance-based control strategies. 

**Figure 21:**
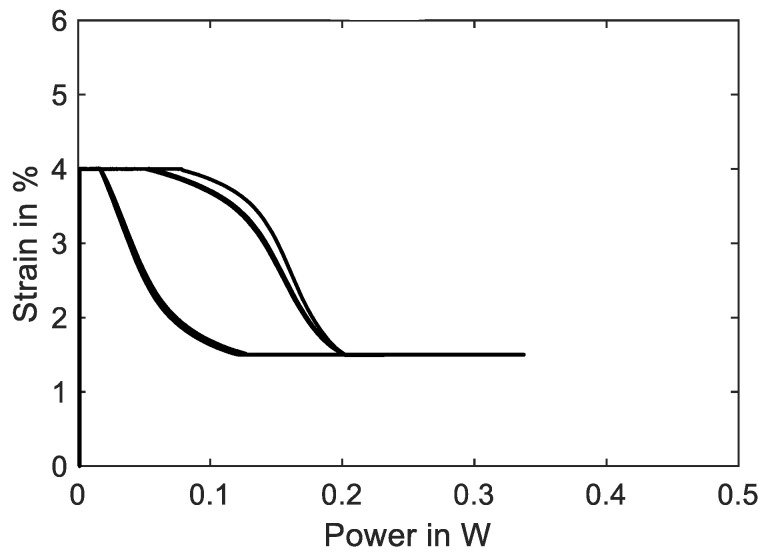
Results of actuator tests with wire specimen “SAES Getters Smartflex” of 100 µm diameter under spring load biasing with end stops and 60 °C ambient temperature. A triangular current signal with an amplitude of 180 mA and a signal duration of 60 s is applied. Three iterations are performed for the experiment. The diagram shows the strain over electrical power behavior.

If these limits can be detected, the overshoot in material stress can be reduced, for example, with a feedback control. This can lead to better fatigue life and higher energy efficiency of SMA systems. When looking at the strain–power hysteresis in Figure 21, compared to Figure 18, it can be observed that it has moved towards the lower left corner and decreased in width and height. Because of the increased ambient temperature, the electrical power needed to start the transformation is reduced to under 0.2 W, and the end stops limit the strain. It is also evident that a maximum current of only about 150 mA is sufficient to reach full contraction in this configuration.

### 3.8. Training—Cyclic Tensile and Actuator Tests

**Figure 22:**
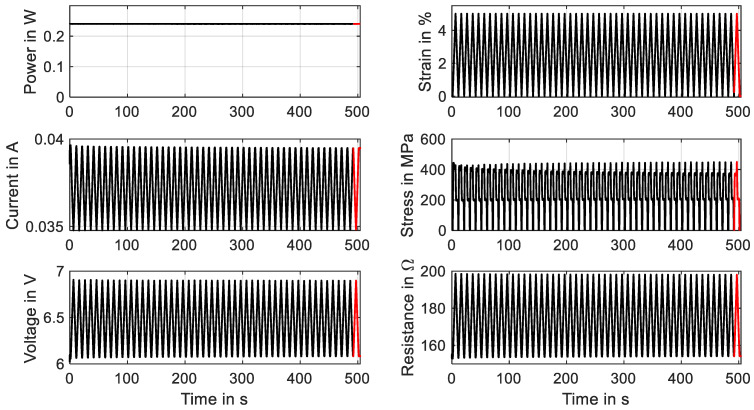
Results of cyclic tensile tests with wire specimen “SAES Getters Smartflex” of 24 µm diameter under constant power heating with 0.23 W, 5% maximum strain and a strain rate of 0.005 s^−1^. The results of 50 cycles for a wire training are plotted over time. The final cycle is plotted in red.

Commercially available NiTi actuator wires are usually trained and exhibit a stable behavior when used within a typical range of 200 MPa. As is shown in Section 3.6 and Section 3.7, excessive material stress leads to instable stress–strain characteristics, and the stroke and force output degrade over time. This aspect and the residual strain can lead to the failure of a system in the very beginning. To avoid this, SMA wires in high-stress applications need to undergo an additional training procedure to achieve a stable and predictable behavior. A distinction can be made between two basic principles of training: thermal cycling and mechanical cycling. To investigate the influence of these training methods with different parameters, the data acquisition of the test rig is designed to handle large data sets of test series with up to 100 cycles. In the following, one example for each training method is described by means of two different wire samples. In Figure 22, the results of a training process of a “SAES Getters Smartflex” NiTi wire with 24 µm are presented by means of mechanical cycling. The heating power is held constant at 0.23 W for the procedure, and 50 tensile tests with a maximum strain of 5% and a strain rate of 0.005 s^−1^ are conducted. 

**Figure 23:**
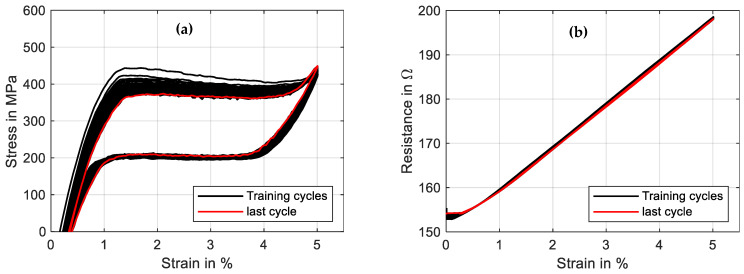
Results of cyclic tensile tests with wire specimen “SAES Getters Smartflex” of 24 µm diameter under constant power heating with 0.23 W, 5% maximum strain and a strain rate of 0.005 s^−1^. The results of 50 cycles for wire training are plotted in a stress–strain plot (**a**) as well as a resistance–strain plot (**b**). The final cycle is plotted in red.

It is observed that over time, the characteristics of stress and resistance change. Starting the experiment, the change is quite pronounced and then leads to a saturation. The stress–strain hysteresis, which is depicted in Figure 23a, undergoes a decrease in hysteresis width, as the upper-plateau stress decreases while the lower-plateau stress stays almost unchanged. Furthermore, the shape of the hysteresis is modified. The negative slope of the upper transformation plateau decreases and is almost constant in the last cycle. Evident is also the residual strain that is increased to 0.45%. In an application, this phenomenon leads to a reduced maximum stroke of the actuator, as is already indicated in Figure 16. The resistance, on the other hand, undergoes less changes. In Figure 23b, the residual strain manifests in a higher minimum resistance (154 Ω instead of 152 Ω), and the overall behavior changes only in a minor way. Parameters that can be varied for a systematic investigation of the training effect are the maximum strain, maximum stress, temperature level (electrical power), strain rate and number of cycles. Wire samples can be compared concerning the evolution of the hysteresis and the residual strain, which can help in the selection of the best-performing wire sample for applications with high material stress and can also give early insights into the fatigue behavior of the wire.

**Figure 24:**
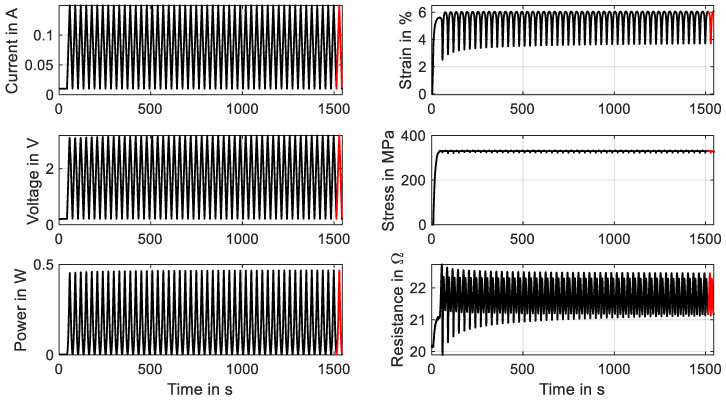
Results of a cyclic actuator test with wire specimen “Fort Wayne Metals NiTi #5” of 73 µm diameter under constant load biasing. A triangular current signal with an amplitude of 160 mA and a signal duration of 30 s is applied. Fifty activation cycles are for the training experiment, of which all results are plotted over time. The final cycle is plotted in red.

The second training method could also be performed with the wire in the actual application and is discussed in the example of a “Fort Wayne Metals NiTi #5” with a diameter of 73 µm and a constant stress of 320 MPa. The triangular current signal has a duration of 30 s and an amplitude of 160 mA. The experiment is performed with 50 cycles, of which the evolution of the results is shown in Figure 24. The main gradient in the characteristics of stress and resistance is visible in the first 10 to 20 cycles. A saturation is visible after that.

**Figure 25:**
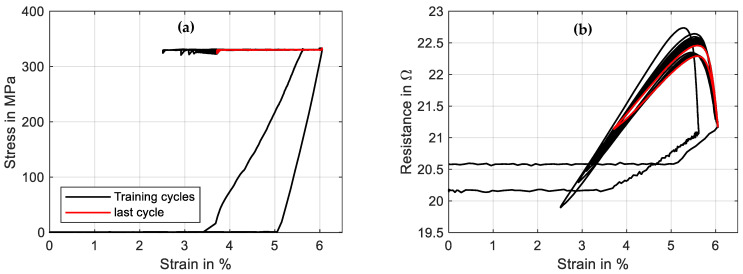
Results of a cyclic actuator test with a wire specimen “Fort Wayne Metals NiTi #5” of 73 µm diameter under constant load biasing. A triangular current signal with an amplitude of 160 mA and a signal duration of 30 s is applied. Fifty activation cycles are conducted for the training experiment, of which the stress–strain diagram is plotted in (**a**), and the resistance–strain behavior is plotted in (**b**). The final cycle is depicted in red.

The change in characteristics of the resistance is more distinct than in mechanical cycling, which can be observed in Figure 25b. The minimum resistance increases from 19.9 Ω to 21.2 Ω, and the hysteretic shape and maximum value change as well.

The actuator stroke decreases from 3.5% strain to 2.3%, which can be observed in Figure 24 and Figure 25a. In this case, investigations into how an increased heating current can reduce the effect are to be conducted. Of interest is also the influence of various stress levels and spring stiffnesses on the stabilization of the material characteristics. For both training methods, the minimum cycles needed for an effective training to new stress levels are of special interest. To evaluate the influence of various training methods and parameters, tensile tests for a basic characterization of the trained wire can be performed. From the outputs, the evolution of important data such as the residual strain, the intrinsic two-way effect and the hysteresis width can be extracted. The actual actuator performance can also be verified with actuator tests after the training series. 

## 4. Conclusions and Outlook

In this work, the design and implementation of a SMA micro-wire characterization test bench are presented. With exemplary measurements of a variety of experiments, the multifunctionality of the setup is shown. On the presented test rig, with its unique properties and features, various application-oriented experiments and tests can be performed. The scope of functions is illustrated with a full range of wire diameters. Meaningful basic characterizations with differently heated tensile tests as well as actuator tests at adjustable ambient temperatures with variable biasing are performed, and the results are presented. Analyzing the results, we can extract, among other parameters, Young’s modulus of martensite and austenite, the hysteresis width, electrical resistivities, residual strains and functional fatigue after different training procedures. The setup helps to design actuator systems for many applications under difficult conditions such as high temperature and high stress. Due to the repeatable and significant results, the setup is well-placed for model and simulation validation. The most important lessons learned during the design and validation of the setup are the importance of reducing the friction with the help of air bearings, the necessity to isolate microwires from the surrounding air, as well as the precision needed to measure low electrical currents. In a future work, a systematic and application-oriented approach to measuring and characterizing a SMA wire sample on this test rig will be presented.

## Data Availability

Data is available within the manuscript.
